# Risk Factors for Sexual Offenses Committed by Men With or Without a Low IQ: An Exploratory Study

**DOI:** 10.3389/fpsyt.2022.820249

**Published:** 2022-04-25

**Authors:** Audrey Vicenzutto, Christian C. Joyal, Émilie Telle, Thierry H. Pham

**Affiliations:** ^1^Forensic Psychology Department, University of Mons (UMONS), Mons, Belgium; ^2^Psychology Department, University of Québec at Trois-Rivières (UQTR), Trois-Rivières, QC, Canada; ^3^International Center of Comparative Criminology, University of Montreal, Montreal, QC, Canada; ^4^Research Center, Philippe Pinel National Institute of Forensic Psychiatry, Montréal, QC, Canada; ^5^Social Defense Research Center (CRDS), Tournai, Belgium

**Keywords:** sexual offense, low IQ, SORAG, RSVP, forensic

## Abstract

Although risk factors associated with offending and recidivism are relatively well-established for mainstream sexual offenses, much less is known about men with a low IQ who have sexually offended (MIQSO), let alone those with forensic involvement. In this exploratory study, 137 convicted for the commission of at least one sexual offense and found not criminally responsible because a mental disorder were recruited in a maximum-security hospital. They were all assessed with the SORAG (static risk factors) and the RSVP (dynamic risk factors). Compared with MIQSO (*N* = 76), men with an average or higher IQ who have sexually offended (MSO, *N* = 61) obtained significantly higher scores on static factors related with general delinquency (histories of alcohol abuse, non-violent criminality, violent criminality, and sexual offense) and dynamic factors related with sexual delinquency, paraphilia, and recidivism (chronicity, psychological coercion, escalation, sexual deviance, and substance abuse). In contrast, MIQSO obtained significantly higher scores on major mental illness, problems with planning and problems with self-awareness. Logistic regressions revealed that both the SORAG and RSVP were useful to predict group membership. It is concluded that risk factors related with general and sexual delinquency better describe offenses committed by MSO, whereas risk factors related with mental disorder, lack of insight and contextual impulsivity better describe offenses committed by MIQSO.

## Introduction

Although the prevalence of men with a low IQ who have sexually offended (MIQSO) is notoriously difficult to ascertain (1–3), the association between lower IQ and higher odds of committing general, violent or sexual criminality is well established (4–6). In fact, people with a low IQ (i.e., a total score of 70 or less) are at increased risk to both commit and being victimized of sexual abuse (7–9). Evaluating and treating patients with a low IQ or an intellectual disability [ID; i.e., having both a low IQ and deficits in adaptive functioning (10)] in forensic settings represent one of the most complex challenge in psychiatry (11), especially among those who have committed a sexual offense (8). Determining the specific (if any) clinical and criminogenic factors of sexual offenses committed by men with a low IQ treated in forensic settings is of utmost importance, not only to better understand the circumstances and motives associated with this particular type of offense, but also for clinicians who have to decide which individual is at risk (or not) to reoffend.

Given the complexity and specific challenges associated with understanding and treating MIQSO, a growing number of handbooks, reviews, and studies have been published during the past decade about the origin, context, assessment, and treatment of this type of sexual offending [e.g., (12–15)]. The main goal of this exploratory study was to collect empirical data concerning psychological and criminogenic aspects of MIQSO recruited in a forensic setting with two risk assessment tools, the Sex Offender Risk Appraisal Guide [SORAG, static risk factors; (16)] and the Risk for Sexual Violence Protocol [RSVP, dynamic risk factors; (17)]. A second objective was to compare these data to those of fellow patients with an average or higher I.Q. who also have committed a sexual offense (MSO) in order to explore the possibility that some factors might help differentiating these groups and better explain the origins and circumstances related with their index crime.

While risk factors associated with sexual offending in general are relatively well established [see (18) for a review], research concerning MIQSO is much more limited because it is hampered with several methodological challenges (3). Possible etiological and contextual factors associated with this type of offense are complex and intricate. On one hand, some of these risk factors appear to be similar to those associated with mainstream sexual offending, i.e., related with delinquency in general [e.g., criminal history, antisocial behaviors, lack of empathy, behavioral impulsivity; see (19, 20), for reviews]. One the other hand, additional (or different) risks factors might be more specifically related to MIQSO, including lower socio-sexual knowledge, less paraphilic interests, lower target specificity, higher prevalence of severe mental disorders, and lower rates of substance abuse. However, conclusions about these unspecific and specific risk factors for MIQSO are still unclear, especially for those treated in forensic settings. Identifying risk factors for sexual offending more specifically related with MIQSO, compared to those of men with an average or higher IQ who have sexually offended (MSO), would help better understanding or characterizing their particular needs. Current knowledge about risk factors concerning MIQSO and assessed in this study is briefly summarized below.

### Childhood Sexual Abuse

Although most victims of child sexual abuse will not sexually offend and most people who sexually offend were not sexually abused, there is a significant link between childhood sexual molestation and subsequent sexual abuse (21). Whereas rates of childhood sexual abuse are also elevated among MIQSO (20), it remains to be seen if these results apply to the forensic population.

### Antisociality and Psychopathy

The prevalence of antisocial behaviors seems to be lower (or at least similar) among MIQSO compared with MSO (22). For instance, histories of nonsexual violent offenses might be significantly lower in MIQSO compared with MSO (23), although this remains to be confirmed. As for psychopathic traits in MIQSO, although data are still scarce, they deserve further investigation (24).

### Poor Impulse Control

Impulsivity and/or personality traits associated with impulsivity (e.g., conduct disorder, antisocial personality disorder) are classically linked to sexual offending in general [e.g., (25)]. Although behavioral impulsivity may also prompt people with a low IQ to commit non-sexual aggressive behaviors (26, 27), the few available comparative studies involving MIQSO have generated mixed results. Glaser and Deane (28) reported that among offenders with ID, those who have committed a sexual offense were significantly less likely to be involved in an offense that required planning (that is, their offenses were more likely to be of the impulsive/reactive type), than those who have committed a non-sexual offense. These authors concluded that sexual offenses committed by persons with ID generally result from impulsivity and poorly controlled behavior rather than an underlying sexual deviance. However, in an attempt to directly test this impulsivity hypothesis, two subsequent studies generated negative results, both based on an adapted version of the Barratt Impulsiveness Scale (BIS). First, Parry and Lindsay (29) reported that compared to non-sex offenders and non-offenders, sex offenders (all with ID) obtained significantly lower scores at the BIS, that is, they were considered as being less impulsive. Second, these results were confirmed by Snoyman and Aicken (30) with an independent sample of participants. The BIS, however, is a self-reported measure not designed to be used in legal settings (it is prone to false negative results) and more sensitive to trait (stable) than state (contextual) impulsivity. Therefore, the possibility remains that a lack of contextual behavioral control plays a significant role in sexual offenses committed by persons with a low IQ.

The possible role of state (vs. trait) impulsivity in the commission of sexual offenses by persons with a low IQ is suggested by indirect evidence. Based on the Ward and Hudson (31) self-regulation pathways model, Lindsay et al. (32) reported that among men with ID, a lower mean IQ (M = 64.5) was associated with higher odds of adopting an approach-automatic pathway of sexual offending, which is associated with poor planning and higher impulsivity [see also (33)]. According to Lindsay (20), these men fail to attempt to inhibit their sexual impulse because they are not sufficiently aware that these acts are socially unacceptable. Indeed, among MIQSO, the approach-automatic pathway is associated with higher rates of recidivism compared with those who adopt the approach-explicit pathway (32). Accordingly, several treatment programs stress the importance of enhancing self-regulation and self-control capacities among MIQSO [e.g., (34)]. However, several MIQSO [e.g., 47% in (35)] adopt the approach-explicit pathway, which is associated with planning, not impulsivity (31) and more serious (with contact) offenses (32). Therefore, consideration for MIQSO with convictions for serious sexual offenses is warranted to explore the influence of contextual impulsivity.

### Insufficient Sexual and Social Knowledge

Traditionally, sexual offenses committed by persons with a lower IQ were viewed as causally linked with sexual naivety, a lack of sexual knowledge and experience, less opportunities for consensual relationships, and low social/interpersonal skills [see the Counterfeit deviance theory for instance; (36, 37)]. These factors, paired with an understandable desire for interpersonal proximity and sexuality by people with low IQ (just like most persons), may indeed contribute to inappropriate sexual behaviors or relatively minor sexual offenses [e.g., improper talking or touching, kissing a stranger, indecent exposure, peeping; (20, 38)]. It is worth noting, however, that several MIQSO (especially recidivists and/or those with serious sexual offenses) possess superior sexual knowledge than non-sexual offenders or non-offenders with a lower IQ [e.g., (39–41)]. Therefore, sexual naivety and lack of knowledge cannot explain most serious cases of sexual offenses committed by MIQSO (20). Again, inclusion of MIQSO convicted of serious sexual offense is warranted to investigate the Counterfeit deviance theory.

### Paraphilic Interests

Among mainstream sexual offenders, interests for paraphilic or illegal sexual behaviors are clear (and rather evident) risk factors for the commission of a corresponding offense, especially in recidivists [e.g., (42)]. Although the same association is commonly hypothesized to apply to MIQSO [i.e., (40, 43, 44)], data are still rare and inconclusive. Distinguishing sexual deviance (interests for illegal sexual behaviors), from immature, inappropriate, impulsive or less targeted behavior associated with intellectual deficits is challenging but crucial in forensic evaluations (37, 45). Although paraphilic interests are occasionally reported in people with developmental disorders [e.g., autistic spectrum disorder; (46–48)], their prevalence is not established in people with low IQ. Individuals with low IQ are not overrepresented among paraphilic sexual offenders (49) and the characteristics of victims of MIQSO are less specific (lower sexual discrimination, with victims of varied age and gender), on average, than those of MSO (23, 38, 50), which argues against the hypothesis of elevated rates of paraphilia among MIQSO. As stressed by Day (51) some time ago, “True sexual deviance is rare. [People with a low IQ] lack the intellectual sophistication and cognitive and imaginative capacity to develop and feed some of the more extreme paraphilias. It is a mistake to conceptualize all sex offending behavior in the learning disabled as aberrant, as is sometimes done.” [p. 280; see also (14)]. Indeed, a recent study failed to find any difference between MIQSO and MSO for interests (including fantasies) in pedophilia, exhibitionism, fetishism, sadomasochism, voyeurism, and rape (22).

Still, studies of sexual interests among persons with low IQ are rare, rendering difficult the estimation of paraphilia rates in this population. The few studies reporting paraphilic fantasies or behaviors among persons with low IQ are generally based on rare or single cases [e.g., (38, 52, 53); see (22) for an exception]. In addition, paraphilic behaviors adopted by people with low IQ usually fail to reach the basic diagnostic criteria for paraphilia [i.e., preferential, intense, and recurrent interests; (37, 54, 55)]. Multidisciplinary evaluation and penile plethysmography also seem to disaffirm diagnoses of paraphilia in sexual offenders with ID (56). A possible exception is pedophilia. Although most available reports are based on acted-out cases of child sexual abuse in which the offender had been apprehended, not necessarily preferential or targeted behaviors (57–59), penile plethysmography studies suggest that some MIQSO indeed show preferential pedophilic interests (23, 57, 60). However, some studies report higher rates of child victims among MIQSO compared with MSO (23), whereas others do not (49). Children victimization from MIQSO may also be due to a lower sexual selectivity, both for age and gender, instead of genuine pedophilia (38, 50). The functional age of the offender may also be causally involved (i.e., attractiveness toward children), as well as general hypersexuality (61) and sexual preoccupations (62). Rates of child sexual offenses or pedophilia may also be inflated in MIQSO due to referential bias (child sexual abuse being more likely to be reported; 19). In any case, it is plausible that past sexual victimization, limited opportunities for consenting sexual relations with adults, circumstantial opportunities with minors, a lack of socio-sexual knowledge (37) or a lower discrimination toward sexual targets [low sexual specificity; (38, 50)], rather than a genuine paraphilic interest, explain most of the commissions of a paraphilic sexual offense by MIQSO. For instance, ranges of victim age and gender are clearly larger for MIQSO than for mainstream sexual offenders [e.g., (43, 59)]. Overall, the link between paraphilia and MIQSO deserves further attention.

### Higher Rates of Psychiatric Comorbidity

Although assessing the presence of a psychiatric diagnosis among persons with a low IQ is challenging (e.g., diagnostic criteria often rely on verbal self-descriptions and insight capacities), estimate rates of comorbidity are significantly elevated in this population (63), ranging from 20to 50% across studies (64, 65). Higher rates of comorbid psychiatric and/or neurodevelopmental disorders are also reported in offenders (sexual or not) with ID (20, 43). Most common comorbid diagnoses include psychotic disorders, ADHD/conduct disorders, depression, autistic spectrum disorders, and personality disorders [in descending order of importance; (66)]. Given that all these disorders are by themselves associated with elevated risk of committing an offense, they might increase or mediate the link between low IQ and offending (64, 67). It is worth noting that all these co-morbid disorders are also associated with behavioral impulsivity (at least in men), which is classically associated with offending [sexual or not; (68, 69)]. It remains to be seen whether the rate of psychiatric comorbidity is higher in MIQSO compared with MSO.

### Less Substance Abuse

Abusing alcohol and other drugs is clearly associated with non-sexual and sexual violence in the general population [e.g., (70)]. Along with delinquency-related factors, substance abuse is also associated with general violence committed by persons with a low IQ (71). Interestingly, however, there are some indications that MIQSO are significantly less likely to use or abuse substances than MSO (22, 28, 38). In these cases, the link between behavioral disinhibition and alcohol or drug consumption would be lower, which deserves further investigation.

### Recidivism

Although sexual recidivism is sometime believed to be elevated in people with ID who have sexual offended (72, 73), conclusions are usually based on follow-ups of small, high-risk samples of individuals without comparison groups [generating high rates of sexual offending, ranging between 30 and 50%; (59, 74, 75)]. When MIQSO are recruited in community services, rates of recidivism are lower [e.g., 21% after 4 years, (76)]. Studies following MIQSO referred to specialized clinics also report lower rates of recidivism. For instance, Rice et al. (23) found a 19% recidivism rate for MIQSO after a follow-up of 12.5 years on average (*N* = 59). Interestingly, Rice and colleagues also found that MIQSO were at significantly lower risk to sexually (and non-sexually) reoffend compared to MSO (*N* = 51, 45%). Fedoroff et al. (46) reported a similar rate of contact (hands-on) sexual recidivism (22%) for MIQSO after a follow-up of 2.5 years on average, as well as Lindsay et al. (77) for a follow-up period up to 13 years (23.9%). These rates are comparable to those of mainstream sexual offenders [i.e., 20% in ten-year follow-ups; (78)]. Therefore, total scores of risk assessment measures for sexual offending should not differ between sexual offenders with vs. those without a low IQ.

An alternative method to evaluate recidivism is to document charges for (or reports of) sexual offenses prior to the index crime. Lindsay et al. (76), for instance, found that 62% of their referrals had a previous conviction or clear documented evidence for a sexual offense [see also (79) for a rate of 78%]. Comparisons of previous official charges between MIQSO and MSO remains to be made.

### Risk Assessment for Sexual Offenders With ID

Although most available instruments to assess risks of committing sexual offenses were developed for mainstream sexual offenders, many are used successfully with violent or sexual offenders with low IQ, both for static [(59, 71, 72, 80, 81); see (82) for a review] and dynamic risk factors [(83), see (84) for a review]. Assessing both static and dynamic risk factors is necessary, as each type explains significant and partly independent parts of the variance (82, 84–86).

Different measures of static risk factors have been used in men with low IQ who have sexually offended [e.g., VRAG, RRASOR, STABLE-2000, Static-99R; e.g., (59, 81, 86–88)]. Some studies generated negative results, however, and finding are mixed with certain scales [e.g., (75, 89), see (85) for a review]. To date, one of the best predictive tool for sexual offenders with low IQ is the SORAG [Sex Offender Risk Appraisal Guide; (16)], which is sensitive to recidivism (89). It consists of 14 items assessing static risk factors associated with sexual and non-sexual criminality. The main difficulty with the SORAG is its dependence on the PCL-R [Psychopathy Checklist-Revised; (90)] and penile plethysmography, both time consuming assessments requiring special training.

Dynamic factors have also been successfully assessed in sexual offenders with low IQ based on such instruments as the ARMIDILO-S [Assessment of Risk Manageability for Individuals with Developmental and Intellectual Limitations who Offend–Sexually; (91)] and the SVR-20 [Sexual Violence Risk; (83)]. The Risk for Sexual Violence Protocol [RSVP; (17)] is another promising tool to evaluate the risk of sexual criminality for individuals with low IQ. An evolved version of the Sexual Violence Risk-20 [SVR-20; (92)] and the Historical Clinical Risk-20 [HCR-20; (93)], the RSVP is currently one of the most widely used instruments for sexual offenders in general, with good to excellent interrater reliability (94, 95). Mostly based on dynamic factors, the RSVP is a set of Structured Professional Judgment (SPJ) guidelines for assessing and managing risk of sexual violence. It consists of 22 risk factors encompassing five domains: sexual violence history, psychological adjustment, mental disorder, social adjustment, and manageability. Although it has not yet been used with MIQSO, the RSVP allows assessing several relevant items, including deficits in sexual knowledge, paraphilic interests, problems with anger and impulsiveness, poor self-regulation, planning deficits, psychopathic personality disorder, major mental disorder, and substance abuse.

We previously showed with an independent forensic sample of Belgian men who have sexually offended that associations between the SORAG and the RSVP scores are only moderate (96). Therefore, these two instruments offer complementary information. The feasibility of using the RSVP with MIQSO remains to be demonstrated.

Because most available studies of risk assessment for MIQSO were not conducted in forensic setting, this study aimed at obtaining and comparing data about static and dynamic risk factors with the SORAG and the RSVP, respectively, with groups of MIQSO and MSO recruited in a forensic setting. Given the exploratory nature of the study, no specific a priori hypotheses were posited, although the presence of general a double dissociation was expected. Whereas MSO should score higher than MIQSO on items related with delinquency (i.e., substance abuse, non-violent criminality, antisocial behaviors), the opposite should be seen for items related with mental illness. In order words, static risk factors should be more closely related with sexual offenses associated with an average or higher IQ whereas dynamic risk factors should be more closely related with sexual offenses associated with a low IQ.

## Method

### Participants

A total of 137 men participated in this study, all recruited in a maximum security psychiatric (forensic) hospital, convicted for the commission of at least one in-person sexual offense with a known victim (i.e., not limited to pornography consumption, luring, scatologia or voyeurism) and found not criminally responsible because a mental disorder rendered them “incapable of controlling their actions” (Belgian Defense Act of 1964).

One subgroup of participants (*N* = 76) had an IQ in the range of intellectual disability (i.e., below 70, as assessed with the French version of the Weschler Adult Intelligence Scales [WAIS; (97); M = 57 ± 7.5], whereas the other subgroup (*N* = 61, M = 86 ± 13.8) had a significantly higher IQ (M-W U = 0, p =0.000, r = 0.86). Given that no validated French version of adaptive behavior measure was available at the time of data collection, group definition was based solely on IQ (not ID).

### Instruments

Based on the institutional files of each participant, age at admission, length of stay, index sexual crime (rape, indecent contact behavior or indecent exposure), and prior sexual and non-sexual crimes (rape, indecent contact behavior, indecent exposure, interpersonal violence, non-sexual non-violent crimes) were registered. In Belgium, rape is defined as a sexual penetration, either vaginal, anal or oral, total or partial, with the penis, fingers or an object, involving a non-consenting person (adult or minor, male or female). Indecent contact behaviors include non-penetrative sexual contacts (i.e., not limited to verbal or Internet offenses) involving a non-consenting, identified person (i.e., not a crowd), including fondling or manipulating the genitals, anus, or the female breast; either directly or using an object, or asking for such contact; caressing or kissing; and forcing to undress or to expose their genitals. Indecent contact behaviors include both violent (with an adult or minor victim) and non-violent (with a minor victim) offenses, but this study does not differentiate them. Although the Belgium law does not include a specific child sexual abuse category, non-violent indecent contact behaviors necessarily involve a minor victim. Indecent exposure is defined as the deliberate act of exposing one's genitals to one or more people (adult or minor, male or female) in a public place, but also as having a consensual sexual behavior in public before unsuspecting strangers. From 2010 to 2020, two-thirds (67%) of rape victims known to the police were adults in Belgium, whereas half of known victims of indecent contact behaviors were minors [15 years of age or less; (98)]. Therefore, indecent contact behaviors are more likely to involve children or young adolescent victims than rape.

In addition, two well-established measures of mainstream sexual recidivism risk were used, the SORAG for static (historical, fixed) factors (16) and the RSVP for more dynamic (fluctuating, manageable) factors (17, 94). This study was conducted for research purposes and based on individual items of each instrument, as the goal was not to assess rates of recidivism or predictive values of the scales. The SORAG is one of the best predictors of future sexual offenses in mainstream sexual offenders [e.g., (99, 100)]. It consists of 14 items assessing static risk factors associated with sexual and non-sexual criminality (Table 1). In this study, phallometric evaluations and PCL-R (psychopathy) scores were available only for a portion of participants. Given that the main goal of the investigation was to assess individual factors of the SORAG (not to predict recidivism), phallometric, and PCL-R results were not included. SORAG scores range from −26 to +51, generating one of nine possible levels of risk (probabilities) for violent and sexual recidivism. Percentile ranks are also obtained, based on a large sample of offenders (16). Interrater reliability is excellent [0.90; (16)] and predictive validity is good for general (AUC = 0.73), sexual (AUC = 0.73) and violent (AUC = 0.76) recidivism (101). In this study, a French version of the SORAG validated with a Belgian sample of sex offenders recruited in a forensic psychiatric setting was used (102). The feasibility of using the SORAG with men having an ID has also been demonstrated (89). The internal consistency of the SORAG was satisfying in this study (Cronbach's alpha: Total score = 0.70).

**Table 1:** Comparisons of scores obtained by participants with lower (50–70) vs. higher (71–110) IQ on the 14 items of the Sex Offender Risk Appraisal Guide (SORAG) (significant between-group differences in bold).

	**MIQSO**		**MSO**		**Statistics**		
		**M (SD)**		**M (SD)**	**M-W U test**	***p***	**Effect size (r)**
**Age (years)**	**45.12 (10.44)**	**49.16 (12.29)**	**1,831**	**0.000**	**0.18**		
Length of stay (years)	13.17 (6.44)	12.29 (6.88)	2,254	0.336			
					Fisher's exact test	*p*	Effect size (Cramer's V)
Index sexual crimes	93.40%	93.40%	0.00	0.999			
Rape	67.10%	57.40%	1.37	0.242			
**Indecent contact behavior**	**53.90%**	**75.40%**	**6.73**	**0.012**	**0.22**		
Indecent exposure	15.80%	26.20%	2.28	0.142			
Prior sexual crimes	35.70%	50.00%	2.21	0.170			
Rape	21.80%	22.00%	0.00	1.00			
Indecent contact behavior	23.20%	38.00%	2.74	0.138			
**Indecent exposure**	**3.60%**	**22.00%**	**8.14**	**0.006**	**0.28**		
**Index violent crimes**	**14.50%**	**30.00%**	**4.82**	**0.035**	**0.19**		
Prior violent crimes	25.50%	30.00%	0.27	0.665			
Index non-sexual non-violent crimes	17.10%	19.70%	0.15	0.824			
Prior non-sexual non-violent crimes	40.00%	52.00%	1.52	0.244			

*MIQSO, Men with low IQ who have sexually offended; MSO, Men with average or higher IQ who have sexually offended; M, Mean; SD, Standard deviation; N, Number of participants with valid data; M-W U, Mann-Whitney U test*.

*Bold: significant between-group differences*.

The RSVP (17, 94) allows assessing 22 risk items for sexual offending, coded as: Yes (definitely present), Maybe (partially or probably present or evidence is inconclusive) or No (definitely absent or no evidence is present). The risk items are grouped into five main factors: Sexual Violence History (Factor A, including five risk items related to the individual's history of sexual violence); Psychological Adjustment (Factor B, including five risk items covering psychological adjustment aspects and have a strong and relatively specific conceptual link to the decisions that lead individuals to engage in sexual violence); Mental Disorders (Factor C, including five risk items which indicate the presence of severe psychopathology); Social Adjustment (Factor D, including four risk items related to relationships with others and social roles and obligations); and Manageability (Factor E, including three risk items that reflect problems in managing the risk of sexual violence in the community). Evaluations are made for three different periods (past, present, and future) for each participant. Although few studies have yet assessed the psychometric qualities of the RSVP, good to excellent inter-rater reliability have been documented for past, recent, and relevance scales, as well as judgment summaries (103). Temporal stability and predictive validity of the scale have also been demonstrated (94, 95). Given the research purposes of this study, the RSVP three-category scoring scale was quantified as no evidence (0), partial evidence (1) and definite evidence (2), with a total score varying from 0 to 44 for each period (past, current, and future). As stressed in the SVR-20 (92) and HCR-20v3 (104) manual, point rating is useful for research purposes. In the present study, the internal consistency of RVSP was acceptable to satisfying for total scores (Cronbach's alpha: Past = 0.69: Current = 0.71; Future = 0.71) and factors in each period (Cronbach's alpha range: Factor A = 0.73–0.78; Factor B = 0.67–0.70; Factor C = 0.62–0.64; Factor D = 0.69–0.76; Factor E = 0.77–0.78).

### Procedure

Participants were evaluated individually at least 1 month after their admission to the facility by psychologists trained in the administration of the instruments, including the first and last authors of this study.

### Data Analyses

#### Bivariate Comparisons

Given the preliminary nature of this study (relatively low number of participants per group) and the importance of avoiding type II statistical errors (not detecting true differences between groups), a series of inter-group comparisons were first conducted with uncorrected probability values (set at 0.05). The first comparisons were performed on mean age, length of stay, index sexual crime (rape, indecent contact behavior or indecent exposure), and prior sexual and non-sexual crimes (rape, indecent contact behavior, indecent exposure, interpersonal violence, non-sexual non-violent crimes).

Then, mean scores at the SORAG and RVSP tools were compared between groups. These exploratory comparisons also served to select potential predictors for subsequent logistic regressions (105). Since most data were not normally distributed (as assessed with the Kolmogorov-Smirnov test), non-parametric Mann-Whitney U or chi-squared tests were used. Effect sizes were also computed (*r* for mean comparisons; Cramer's *V* for Chi-squares) with sizes of 0.10, 0.30, and 0.50 considered small, medium, and large, respectively (106).

#### Binary Logistic Regressions

A second wave of analyses was conducted after logarithmic transformations of the data with two binary logistic regressions (one for each risk assessment measure; backward stepwise approach) to evaluate the relative value of significant SORAG and RSVP factors (as assessed with the between group comparisons) to predict group membership (MIQSO vs. MSO). A stop rule at 0.01 was applied in order to limit type I statistical error. The choice to perform a logistic regression for each instrument is justified by their shared variance (e.g., *History of alcohol problems* in SORAG and *Problems with substance use* in RSVP). In addition, the instruments present conceptual differences with regard to their background, main objective, inclusion of static vs. dynamic risk factors, temporality, and scoring. The SORAG is based on an actuarial approach (correlations with recidivism, empirically based), including static and historical risk factors. Its items are rated on a continuum of values ranging from negative to positive. The RSVP is based on more dynamic factors and clinical measures in addition to static factors, derived from an experiential basis (experts' advice). Its items are assessed on a gradation of value varying from 0 to 2. It is assessed dynamically with the possibility of repeating assessments, integrating the concept of change, which the SORAG does not. The RSVP is also intended to manage risk in the short and medium terms, whereas the SORAG serves to evaluate long-term risks. Based on the sample size, a maximum of 16 predictors can be included (105), five for the SORAG and 11 for the RSVP. The major mental illness item was not considered because the RSVP definition includes low IQ, which is the independent variable (definition of the groups) of this study. We verified multi-collinearity of potential predictors using variance inflation factors (VIF) and its reciprocal, tolerance statistics. However, there is little consensus in the literature on their interpretation. A VIF >10 (or a tolerance lesser than 0.10) indicates a serious problem of multi-collinearity [(107, 108); cited by (105)]. For SORAG items, VIF indicated a low probability of collinearity between the predictors (VIF = 1.08–1.58; tolerance = 0.63–0.92). This was also the case for the RSVP (VIF = 1.26–3.76; tolerance = 0.27–0.83). The backward method including the Wald statistic (Chi-square Wald) was selected due to the small sample size. This method allows the associated effect of the set of potential predictors to be represented until the analysis is refined toward the most significant predictors. The contribution of the backward models was controlled by running forward regression models (not reported here), which confirmed the robustness of the final models identified by the backward regression. The analysis removes, model by model, variables with Wald statistic lower than 0.10 (105). The Wald statistic controls for the contribution of each predictor to the model. Odds ratio (Exp B) were also used, corresponding to the exponential of coefficient B and indicating changes (significant or not) in proportions. Finally, explained variance was estimated with *Nagelkerke's R*^2^ transformations (105).

### Ethical Considerations

This study was approved by the research ethical committee of the forensic psychiatric hospital and its procedures followed the ethical principles of the Helsinki declaration. All participants were volunteers and did not receive any financial compensation. They signed an informed consent form specifying the purpose of the study and guaranteed anonymity (no identification information appeared in the data set).

## Results

MIQSO were significantly younger, on average, and significantly less likely than MSO to have committed an indecent contact behavior or an additional nonsexual violent act as index crime (Table 1). MIQSO were also significantly less likely than MSO to have committed prior indecent exposure than MSO. Approximately a third of MIQSO (35.7%) and half of MSO (50%) were sexual recidivists, i.e., they had committed at least one known sexual offense prior to the index crime. These rates were not significantly different, although this might be due to a lack of statistical power.

As shown in Table 2, MIQSO scored significantly lower than MSO for histories of alcohol abuse, non-violent criminality, violent criminality, and sexual offense at the SORAG. Significantly fewer MIQSO than MSO have been married, whereas both groups obtained similar mean scores for the presence of a personality disorder (Table 2).

**Table 2:** Comparisons of scores obtained by MIQSO and MSO on the 14 items of the sex offender risk appraisal guide (SORAG).

	**MIQSO**		**MSO**		**Statistics**		
	***N***	**M (SD)**	***N***	**M (SD)**	**M-W *U*-test**	***p***	**Effect size (r)**
Lived with both parents to age 16 (R)	76	0.43 (2.52)	61	0.87 (2.49)	2,116.50	0.313	
Elementary school maladjustment	72	0.88 (2.22)	60	0.85 (2.15)	2,159.50	0.998	
**History of alcohol problems**	**75**	**−0.12 (0.85)**	**60**	**0.30 (1.08)**	**1,779.00**	**0.028**	**−0.19**
**Never married**	**76**	**0.61 (1.02)**	**61**	**−0.43 (1.51)**	**1,521.00**	**0.000**	**−0.38**
**Non-violent criminal history**	**71**	**−0.82 (1.88)**	**59**	**0.22 (2.22)**	**1,547.50**	**0.004**	**−0.25**
**Violent criminal history**	**75**	**1.65 (3.30)**	**60**	**3.07 (3.39)**	**1,771.00**	**0.020**	**−0.20**
**Convictions for prior sex offenses**	**76**	**−0.08 (1.40)**	**61**	**0.84 (1.98)**	**1,685.00**	**0.002**	**−0.26**
History of sex offenses against girls only (R)	75	2.93 (1.78)	60	2.80 (1.85)	2,175.00	0.670	
Failure on prior conditional release	72	1.50 (1.51)	60	1.75 (1.49)	1,980.00	0.341	
Age at index offense (R)	76	−1.20 (2.64)	60	−1.90 (2.90)	1,945.00	0.128	
DSM-III personality disorder	71	1.03 (2.46)	59	1.39 (2.36)	1,943.00	0.395	
DSM-III schizophrenia	76	0.47 (1.36)	58	0.38 (1.46)	2,152.00	0.699	
Total score	76	0.47 (1.36)	61	9.92 (11.11)	1,915.50	0.081	

*MIQSO, Men with low IQ who have sexually offended; MSO, Men with average or higher IQ who have sexually offended; M, Mean; SD, Standard deviation; (R), reversed item; N, Number of participants with valid data; M-W U, Mann-Whitney U-test; Bold, significant between-group differences*.

As shown in Table 3, MIQSO obtained significantly lower scores than MSO on the Sexual Violence History (Factor A) of the RSVP, but only for the future period. In contrast, MIQSO obtained significantly higher scores than MSO for past, current, and future Mental Disorder (Factor C). No difference emerged between the groups for Psychological Adjustment (Factor B), Social Adjustment (Factor D), and Manageability (Factor E).

**Table 3:** Comparisons of scores obtained by MIQSO and MSO on the 22 items of the risk for sexual violence protocol (RSVP) for current time.

	**MIQSO**		**MSO**		**Statistics**		
	***N***	**M (SD)**	***N***	**M (SD)**	**M-W *U*-test**	***p***	**Effect size (r)**
Factor A—sexual violence history	70	4.01 (2.80)	57	4.70 (2.7)	1,723.50	0.184	
**Chronicity of sexual violence**	**72**	**1.10 (0.97)**	**54**	**1.42 (0.90)**	**1,691.50**	**0.047**	**−0.18**
Diversity of sexual violence	72	0.54 (0.85)	57	0.49 (0.87)	1,964.00	0.594	
Escalation of sexual violence	72	0.53 (0.85)	57	0.74 (0.92)	1,801.50	0.157	
Physical coercion in sexual violence	71	0.96 (0.96)	57	0.74 (0.94)	1,780.50	0.190	
**Psychological coercion in sexual violence**	**71**	**0.89 (0.96)**	**57**	**1.32 (0.89)**	**1,553.50**	**0.012**	**−0.22**
Factor B—psychological adjustment	66	6.71 (2.17)	55	6.56 (2.02)	1,717.50	0.607	
Extreme minimization or denial of sexual violence	72	1.50 (0.77)	56	1.43 (0.81)	1,926.00	0.610	
Attitudes that support or condone sexual violence	71	0.89 (0.95)	56	0.89 (0.95)	1,981.00	0.970	
Problems with self-awareness	72	1.79 (0.53)	56	1.75 (0.64)	2,013.00	0.981	
Problems with stress or coping	72	1.60 (0.74)	56	1.59 (0.76)	2,012.00	0.980	
Problems resulting from child abuse	67	1.09 (0.88)	55	0.91 (0.97)	1,651.50	0.285	
**Factor C—mental disorder**	**68**	**5.10 (1.95)**	**54**	**4.20 (1.78)**	**1,389.00**	**0.018**	**0.21**
Sexual deviance	70	1.09 (0.93)	56	1.23 (0.87)	1,800.50	0.392	
Psychopathic personality disorder	70	0.20 (0.55)	55	0.16 (0.46)	1,914.00	0.925	
**Major mental illness**	**71**	**2.00 (0.00)**	**57**	**1.11 (0.99)**	**1,100.50**	**0.000**	**0.56**
Problems with substance use	71	0.97 (0.97)	57	1.21 (0.98)	1,766.50	0.163	
Violent or suicidal ideation	70	0.77 (0.93)	57	0.60 (0.88)	1,802.00	0.276	
Factor D—social adjustment	65	5.60 (1.80)	56	5.62 (1.83)	1,811.50	0.964	
Problems with intimate relationships	67	1.79 (0.54)	57	1.88 (0.43)	1,792.00	0.298	
Problems with non-intimate relationships	70	1.26 (0.85)	57	1.40 (0.82)	1,802.00	0.297	
Problems with employment	69	1.43 (0.85)	56	1.36 (0.86)	1,832.00	0.559	
Non-sexual criminality	71	1.03 (0.97)	57	1.02 (0.97)	2,012.00	0.951	
Factor E—manageability	69	3.61 (1.63)	57	3.79 (1.84)	1,821.00	0.467	
Problems with planning	69	1.70 (0.60)	57	1.56 (0.73)	1,814.50	0.333	
Problems with treatment	71	0.87 (0.86)	57	1.07 (0.92)	1,786.00	0.221	
Problems with supervision	71	1.07 (0.90)	57	1.16 (0.88)	1,918.50	0.587	
Total score	57	25.1 (6.4)	52	24.71 (6.7)	1,441.00	0.803	
Factor A—sexual violence history	69	1.64 (2.23)	57	2.26 (2.90)	1,816.00	0.423	
Chronicity of sexual violence	71	0.52 (0.81)	57	0.60 (0.90)	1,982.00	0.808	
Diversity of sexual violence		70	0.10 (0.35)	57	0.18 (0.57)	1,978.52	
Escalation of sexual violence	71	0.24 (0.60)	57	0.37 (0.75)	1,897.50	0.366	
Physical coercion in sexual violence	71	0.35 (0.68)	57	0.35 (0.72)	1,985.00	0.801	
**Psychological coercion in sexual violence**	**72**	**0.43 (0.75)**	**57**	**0.77 (0.94)**	**1,690.00**	**0.040**	**−0.18**
Factor B—psychological adjustment	65	5.69 (2.23)	55	5.58 (2.28)	1,681.00	0.571	
Extreme minimization or denial of sexual violence	71	1.21 (0.81)	56	1.13 (0.92)	1,907.00	0.672	
Attitudes that support or condone sexual violence	71	0.63 (0.87)	56	0.75 (0.90)	1,852.00	0.452	
Problems with self-awareness	72	1.63 (0.70)	56	1.52 (0.76)	1,872.00	0.381	
Problems with stress or coping	72	1.46 (0.79)	56	1.38 (0.84)	1,926.00	0.616	
Problems resulting from child abuse	67	0.91 (0.85)	55	0.84 (0.94)	1,740.50	0.571	
Factor C—mental disorder	68	3.93 (1.70)	53	3.32 (1.95)	1,525.50	0.139	
Sexual deviance	70	0.86 (0.87)	55	1.15 (0.89)	1,588.00	0.072	
Psychopathic personality disorder	70	0.17 (0.51)	55	0.18 (0.51)	1,902.00	0.839	
**Major mental illness**	**71**	**2.00 (0.00)**	**57**	**0.98 (1.01)**	**994.00**	**0.000**	**0.60**
**Problems with substance use**	**71**	**0.31 (0.69)**	**57**	**0.60 (0.82)**	**1,634.00**	**0.017**	**−0.21**
Violent or suicidal ideation	70	0.56 (0.82)	57	0.42 (0.73)	1,847.00	0.381	
Factor D—social adjustment	64	4.19 (2.19)	56	3.82 (2.17)	1,648.50	0.444	
Problems with intimate relationships	67	1.52 (0.75)	57	1.47 (0.85)	1,909.00	0.998	
Problems with non-intimate relationships	69	1.00 (0.86)	57	1.14 (0.89)	1,790.50	0.356	
Problems with employment	69	1.20 (0.92)	56	0.91 (0.92)	1,607.00	0.078	
Non-sexual criminality	71	0.38 (0.72)	57	0.25 (0.61)	1,856.00	0.252	
Factor E—manageability	68	2.84 (1.62)	57	2.58 (1.74)	1,812.50	0.526	
**Problems with planning**	**68**	**1.60 (0.67)**	**57**	**1.21 (0.86)**	**1,463.50**	**0.007**	**0.20**
Problems with treatment	70	0.61 (0.79)	57	0.68 (0.83)	1,914.00	0.661	
Problems with supervision	70	0.61 (0.79)	57	0.68 (0.83)	1,914.00	0.661	
Total score	54	18.4 (7.2)	51	17.96 (7.08)	1,342.00	0.822	
**Factor A—sexual violence history**	**69**	**2.64 (2.15)**	**57**	**4.19 (2.33)**	**1,261.50**	**0.0001**	**−0.31**
**Chronicity of sexual violence**	**70**	**0.71 (0.66)**	**57**	**1.09 (0.71)**	**1,440.00**	**0.003**	**−0.26**
Diversity of sexual violence	70	0.46 (0.63)	57	0.61 (0.73)	1,779.00	0.234	
**Escalation of sexual violence**	**71**	**0.38 (0.49)**	**57**	**0.77 (0.73)**	**1,451.00**	**0.002**	**−0.27**
Physical coercion in sexual violence	71	0.55 (0.58)	57	0.65 (0.69)	1,903.50	0.521	
**Psychological coercion in sexual violence**	**72**	**0.53 (0.65)**	**57**	**1.07 (0.75)**	**1,257.00**	**0.0001**	**−0.36**
Factor B—psychological adjustment	65	5.54 (2.07)	55	5.45 (2.20)	1,681.50	0.572	
Extreme minimization or denial of sexual violence	72	1.14 (0.76)	56	1.09 (0.81)	1,956.00	0.759	
Attitudes that support or condone sexual violence	71	0.62 (0.74)	56	0.79 (0.82)	1,781.00	0.272	
**Problems with self-awareness**	**72**	**1.65 (0.61)**	**56**	**1.36 (0.73)**	**1,625.00**	**0.027**	**0.20**
Problems with stress or coping	72	1.43 (0.73)	56	1.34 (0.74)	1,876.50	0.456	
Problems resulting from child abuse	66	0.86 (0.82)	55	0.87 (0.88)	1,813.50	0.993	
**Factor C—mental disorder**	**66**	**4.26 (1.49)**	**53**	**3.62 (1.79)**	**1,384.00**	**0.047**	**0.18**
**Sexual deviance**	**68**	**0.85 (0.81)**	**55**	**1.18 (0.80)**	**1,460.00**	**0.027**	**−0.20**
Psychopathic personality disorder	70	0.19 (0.52)	55	0.24 (0.54)	1,829.00	0.444	
**Major mental illness**	**71**	**2.00 (0.00)**	**57**	**0.96 (0.92)**	**816.50**	**0.0001**	**0.66**
Problems with substance use	71	0.62 (0.72)	57	0.79 (0.77)	1,782.00	0.207	
Violent or suicidal ideation	70	0.54 (0.69)	57	0.51 (0.60)	1,991.00	0.982	
Factor D—social adjustment	65	4.55 (1.80)	54	4.29 (1.70)	1,618.00	0.456	
Problems with intimate relationships	67	1.55 (0.58)	57	1.44 (0.65)	1,747.50	0.354	
Problems with non-intimate relationships	70	1.03 (0.74)	57	1.07 (0.75)	1,934.00	0.751	
Problems with employment	69	1.29 (0.79)	55	1.11 (0.68)	1,612.50	0.123	
Non-sexual criminality	0.59 (0.75)	56	0.61 (0.62)	1,855.00	0.567		
Factor E—manageability	68	3.01 (1.47)	57	3.05 (1.42)	1,861.50	0.697	
**Problems with planning**	**69**	**1.57 (0.65)**	**57**	**1.33 (0.66)**	**1,570.50**	**0.028**	**0.19**
Problems with treatment	71	0.73 (0.70)	57	0.91 (0.71)	1,748.5	0.152	
Problems with supervision	70	0.74 (0.69)	57	0.81 (0.64)	1,875.00	0.521	
Total score	53	20.07 (6.04)	49	20.90 (7.07)	1,230.50	0.648	

*MIQSO, Men with low IQ who have sexually offended; MSO, Men with average or higher IQ who have sexually offended; M, Mean; SD, Standard deviation; N, Number of participants with valid data; M-W U, Mann-Whitney U-test; Bold, significant between-group differences*.

Several significant differences emerged between the groups for individual items of the RSVP. MIQSO obtained significantly lower scores than MSO for past and future chronicity of sexual violence; past, current, and future psychological coercion in sexual violence; future escalation of sexual violence; future sexual deviance; and current substance abuse (Table 3). By contrast, MIQSO obtained significantly higher scores than MSO for past, current, and future presence of a major mental illness, current and future problems with planning and future problems with self-awareness (Table 3). No difference emerged between the groups for child abuse and diversity of sexual criminality (past, current or future), whereas current sexual deviance tended (*p* = 0.072) to better characterize MSO.

Based on these results, five significant SORAG variables (between-group differences) were entered in the first logistic regression (history of alcohol abuse, never married, history of non-violent criminality, history of violent criminality, and prior convictions for sexual offenses). Together, these factors significantly predicted group membership (the most in favor of MSO), explaining 28% of the variance (initial model: R^2^ = 0.28; *X*^2^ = 30.42; *p* < 0.001; Table 4). However, only one variable stands out as the most important and significant predictor of the MIQSO membership, regardless of the regression's step (Never married, Exp (B) = 1.77, *p* < 0.001; Table 4). The main effect of this predictor is attenuated by non-violent and sexual offense history, although these factors alone are not statistically significant. The stepwise analysis also shows that a history of violent offenses and a history of alcohol problems, although included in significant predictive models, are hardly contributing to group membership. Indeed, explained variance only slightly decrease after their removal from different models (Table 4).

**Table 4:** Backward stepwise logistic regression coefficients for significant (between groups) items of the sex offender risk appraisal guide (SORAG) to predict group membership.

**Predictors**	**Group**	**Exp (B)**	**CI Exp (B)**	**Standard error**	**Chi-Square wald**	***p***	***R^**2**^***
Initial model^*^							
History of alcohol problems		0.83	0.54–1.27	0.22	0.72	0.396	**0.28**
**Never married**	MIQSO	**1.77**	**1.30–2.41**	**0.16**	**13.24**	**0.000**	
Non-violent criminal history		0.85	0.70–1.04	0.10	2.41	0.121	
Violent criminal history		0.98	0.85–1.13	0.07	0.09	0.762	
Convictions for prior sex offenses		0.81	0.61–1.08	0.14	2.02	0.156	
Step 1^*^							
History of alcohol problems		0.82	0.54–1.26	0.21	0.80	0.372	**0.28**
**Never married**	MIQSO	**1.77**	**1.30–2.42**	**0.16**	**13.33**	**0.000**	
Non-violent criminal history		0.85	0.70–1.03	0.10	2.61	0.106	
Convictions for prior sex offenses		0.79	0.62–1.08	0.13	3.28	0.070	
Step 2^*^							
**Never married**		**1.84**	**1.36–2.48**	**0.15**	**15.69**	**0.000**	**0.27**
Non-violent criminal history	MIQSO	0.84	0.69–1.02	0.10	2.98	0.084	
Convictions for prior sex offenses		0.78	0.61–1.01	0.13	3.65	0.056	

* *Initial model: χ^2^ = 30.42; p = 0.000; Step 1: χ^2^ = 30.32; p = 0.000; Step 2: χ^2^ = 29.53; p = 0.000*.

*MIQSO, Men with low IQ who have sexually offended; Exp (B), exponential of coefficient B, odds ratio; CI, Confidence Interval; R^2^, Nagelkerke R^2^, estimated explained variance; Bold, significant predictors and models*.

As for the second logistic regression, it was based on the eleven significant single items of the RSVP (between-group differences): chronicity of sexual violence (past and future); escalation of sexual violence (future); psychological coercion in sexual violence (past, current and future); problems with self-awareness (future); sexual deviance (future); problems with substance use (current) and problems with self-awareness (future) and planning (current and future). Together, these factors explained 37% of the variance (initial model: R^2^ =0.37; *X*^2^ = 39.06; *p* < 0.001; Table 5) while none of the factors individually is significant (set at 0.01), except for psychological coercion in sexual violence (future) for MSO. Furthermore, the analysis from the initial model to step 3 highlights the non-contribution of variables such as chronicity of sexual violence (past and future) and psychological coercion in sexual violent (current). Through steps 4 to 8, three single predictors were significant or approached significance: escalation of violence (future) and psychological coercion in sexual violence (future) for MSO and problems with planning (current) for MIQSO (Table 5). The final model (Step 8) contained these three significant predictors explaining 32% of the variance (*X*^2^ = 32.08; *p* < 0.001; Table 5). The contribution of the other non-significant factors present in the different models reinforces the effect of the three main predictors because the explained variance decreases following their removal from the fourth step.

**Table 5:** Backward stepwise logistic regression coefficients for significant (between groups) items of the risk for sexual violence protocol (RSVP) to predict group membership.

**Predictors**	**Group**	**Exp (B)**	**CI (Exp B)**	**Standard Error**	**Chi-Square Wald**	***p***	***R^**2**^***
Initial model^*^							
Chronicity of sexual violence (past)	MIQSO	0.96	0.52–1.77	0.31	0.02	0.901	**0.37**
Chronicity of sexual violence (future)		0.87	0.37–2.06	0.44	0.10	0.756	
Escalation of sexual violence (future)		0.46	0.21–1.03	0.41	3.59	0.058	
Psychological coercion in sexual violence (past)		1.57	0.78–3.17	0.36	1.58	0.208	
Psychological coercion in sexual violence (current)		1.14	0.55–2.38	0.37	0.13	0.717	
**Psychological coercion in sexual violence (future)**		**0.23**	**0.08–0.65**	**0.52**	**7.84**	**0.005**	
Problems with self-awareness (future)		1.60	0.82–3.10	0.34	1.93	0.164	
Sexual deviance (future)		0.65	0.36–1.17	0.30	2.03	0.154	
Problems with substance use (current)		0.71	0.39–1.36	0.32	1.01	0.314	
Problems with planning (current)		3.26	1.12–9.53	0.55	4.67	0.031	
Problems with planning (future)		0.60	0.18–1.97	0.60	0.71	0.399	
Step 1^*^							
Chronicity of sexual violence (future)	MIQSO	0.85	0.41–1.73	0.37	0.21	0.649	**0.37**
Escalation of sexual violence (future)		0.46	0.21–1.03	0.41	3.58	0.059	
Psychological coercion in sexual violence (past)		1.56	0.78–3.15	0.36	1.57	0.210	
Psychological coercion in sexual violence (current)		1.16	0.57–2.36	0.36	0.16	0.688	
**Psychological coercion in sexual violence (future)**		**0.23**	**0.08–0.65**	**0.52**	**7.82**	**0.005**	
Problems with self-awareness (future)		1.61	0.83–3.11	0.34	2.01	0.156	
Sexual deviance (future)		0.65	0.36–1.15	0.30	2.17	0.140	
Problems with substance use (current)		0.73	0.39–1.36	0.32	1.00	0.317	
Problems with planning (current)		3.27	1.12–9.55	0.55	4.69	0.030	
Problems with planning (future)		0.60	0.18–1.96	0.60	0.72	0.395	
Step 2^*^							
Chronicity of sexual violence (future)	MIQSO	0.83	0.41–1.68	0.36	0.27	0.601	**0.37**
Escalation of sexual violence (future)		0.46	0.21–1.02	0.40	3.67	0.055	
Psychological coercion in sexual violence (past)		1.60	0.79–3.20	0.36	1.73	0.189	
**Psychological coercion in sexual violence (future)**		**0.25**	**0.10–0.65**	**0.48**	**8.22**	**0.004**	
Problems with self-awareness (future)		1.61	0.83–3.11	0.34	2.00	0.157	
Sexual deviance (future)		0.66	0.37–1.17	0.29	2.06	0.151	
Problems with substance use (current)		0.76	0.42–1.37	0.30	0.85	0.357	
Problems with planning (current)		3.39	1.18–9.73	0.54	5.13	0.024	
Problems with planning (future)		0.58	0.18–1.86	0.60	0.84	0.359	
Step 3^*^							
Escalation of sexual violence (future)	MIQSO	0.43	0.20–0.89	0.38	5.13	0.024	**0.37**
Psychological coercion in sexual violence (past)		1.59	0.79–3.19	0.35	1.72	0.189	
**Psychological coercion in sexual violence (future)**		**0.24**	**0.10–0.61**	**0.47**	**9.00**	**0.003**	
Problems with self-awareness (future)		1.62	0.84–3.13	0.34	2.04	0.153	
Sexual deviance (future)		0.64	0.36–1.12	0.29	2.45	0.118	
Problems with substance use (current)		0.75	0.42–1.37	0.30	0.86	0.355	
Problems with planning (current)		3.49	1.22–9.98	0.54	5.42	0.020	
Problems with planning (future)		0.54	0.17–1.76	0.59	1.00	0.318	
Step 4^*^							
Escalation of sexual violence (future)	MIQSO	0.40	0.19–0.83	0.37	6.09	0.014	**0.36**
Psychological coercion in sexual violence (past)		1.52	0.77–3.00	0.35	1.44	0.229	
**Psychological coercion in sexual violence (future)**		**0.24**	**0.10–0.60**	**0.47**	**9.31**	**0.002**	
Problems with self-awareness (future)		1.57	0.82–3.00	0.33	1.85	0.173	
Sexual deviance (future)		0.65	0.37–1.14	0.28	2.28	0.131	
Problems with planning (current)		3.42	1.22–9.61	0.53	5.44	0.020	
Problems with planning (future)		0.58	0.18–1.80	0.58	0.90	0.342	
Step 5^*^							
**Escalation of sexual violence (future)**	MIQSO	**0.39**	**0.19–0.80**	**0.37**	**6.62**	**0.010**	**0.36**
Psychological coercion in sexual violence (past)		1.51	0.77–2.95	0.34	1.43	0.232	
**Psychological coercion in sexual violence (future)**		**0.24**	**0.10–0.60**	**0.45**	**9.27**	**0.002**	
Problems with self-awareness (future)		1.55	0.81–2.95	0.33	1.76	0.184	
Sexual deviance (future)		0.69	0.40–1.19	0.28	1.81	0.178	
**Problems with planning (current)**		**2.29**	**1.27–4.14**	**0.30**	**7.56**	**0.006**	
Step 6^*^							
Escalation of sexual violence (future)	MIQSO	0.42	0.21–0.84	0.36	5.97	0.015	**0.34**
**Psychological coercion in sexual violence (future)**		**0.36**	**0.19–0.67**	**0.31**	**10.62**	**0.001**	
Problems with self-awareness (future)		1.51	0.80–2.85	0.32	1.61	0.205	
Sexual deviance (future)		0.68	0.39–1.17	0.28	1.95	0.162	
**Problems with planning (current)**		**2.21**	**1.23–3.96**	**0.30**	**7.11**	**0.008**	
Step 7^*^							
Escalation of sexual violence (future)	MIQSO	0.42	0.21–0.84	0.36	5.94	0.015	**0.33**
**Psychological coercion in sexual violence (future)**		**0.36**	**0.20–0.67**	**0.31**	**10.68**	**0.001**	
Sexual deviance (future)		0.71	0.41–1.21	0.27	1.61	0.205	
**Problems with planning (current)**		**2.47**	**1.41–4.34**	**0.29**	**9.90**	**0.002**	
Step 8^*^							
**Escalation of sexual violence (future)**	MIQSO	**0.40**	**0.20–0.81**	**0.36**	**6.57**	**0.010**	**0.32**
**Psychological coercion in sexual violence (future)**		**0.34**	**0.19–0.61**	**0.30**	**13.03**	**0.000**	
**Problems with planning (current)**		**2.45**	**1.39–4.32**	**0.29**	**9.65**	**0.002**	

* *Initial model (χ^2^ = 39.06; p = 0.000); Step 1 (χ^2^ = 39.04; p = 0.000); Step 2 (χ^2^ = 38.88; p = 0.000); Step 3 (χ^2^ = 38.61; p = 0.000); Step 4 (χ^2^ = 37.74; p = 0.000); Step 5 (χ^2^ = 36.81; p = 0.000); Step 6 (χ^2^ = 35.32; p = 0.000); Step 7 (χ^2^ = 33.70; p = 0.000); Step 8 (χ^2^ = 32.08; p = 0.000)*.

*MIQSO, Men with low IQ who have sexually offended; Exp (B), exponential of coefficient B, odds ratio; CI, Confidence Interval; R^2^, Nagelkerke R^2^, estimated explained variance; Bold, significant predictors and models*.

## Discussion

The main goal of this study was to compare men who have committed a sexual offense and admitted in a forensic hospital, with or without a low IQ, with regard to clinical and criminogenic factors associated with sexual recidivism. Concerning static risk factors, MIQSO obtained significantly lower mean scores on general delinquency items of the SORAG than MSO (histories of alcohol abuse and non-violent, violent and sexual criminality). Therefore, static risk factors associated with delinquency seem to be more useful for MSO than MIQSO. These factors, however, only explained 28% of the variance between the groups. Therefore, it is concluded that static risk factors for sexual recidivism are weak and insufficient to discriminate between MIQSO and MSO.

As for dynamic risk factors, MSO obtained significantly higher scores than MIQSO on RVSP items traditionally associated with increased severity of illegal sexual behaviors and odds of recidivism (chronicity, psychological coercion, escalation, sexual deviance, and substance abuse). Intriguingly, these risk factors are also associated with paraphilic offenses [e.g., pedophilia, coercive sadism; (109, 110)]. Given that both RSVP criteria for psychological coercion and this study's definition of indecent contact behaviors include child molestation (both with higher rates in MSO), the question arises as to whether MSO are more likely than MIQSO to victimize children (or to have paraphilic preferences). Given the lack of data in this study, these possibilities deserve further investigation.

In contrast, MIQSO scored significantly higher than MSO on the major mental illness item of the RSVP, suggesting that dynamic factors related with major mental disorders (e.g., psychiatric symptoms, lack of insight) are more relevant to this group for intervention and treatment plans. The major mental illness item is defined as a substantial impairment of cognition, affect, or behavior, which is rather large (17). Although the RSVP definition of major mental illness includes learning disability or low IQ (which serves to define our groups of participants), previous studies suggested that symptoms of co-morbid major mental disorders play a significant role in sexual offenses committed by MIQSO (20, 43). Future investigations with MIQSO should consider major mental disorders and low IQ separately.

MIQSO also scored significantly higher than MSO on problems with self-awareness and problems with planning as assessed with the RSVP. Problems with self-awareness refer to such factors as lack of insight, impaired meta-cognition, deficits in sexual knowledge, and impulsiveness (17, 94), all associated with low IQ and/or major mental disorders. Problems with planning refer to significant difficulties in making and implementing life plans due to such factors as poor self-management and, again, both impulsivity and low self-awareness (17, 94). Therefore, these two RSVP items are not only closely related but they also heavily depend on behavioral or contextual impulsivity. These results are in accordance with the hypothesis that MIQSO, on average, fail to inhibit (or attempt to inhibit) their sexual impulse (20). They also concord with treatment programs aiming at enhancing self-regulation and self-control capacities among MIQSO [e.g., (34)]. Still, further studies should include neuropsychological assessment of contextual (state) impulsivity to confirm these results.

Contrary to expectation (see the introduction), the diversity of sexual offenses was not higher in MIQSO compared with MSO. However, this result might reflect a ceiling effect due to the relatively low level of diversity in both groups in this study. Sexual deviance tended to be more prevalent in MSO than in MIQSO. This item is defined as sexual interest, preference, arousal or behavior focussing on “inappropriate” persons or objects, which may include children (17). This finding, along with higher rates of psychological coercion and index crime associated with children victim (indecent contact behavior) could suggest that MSO might be more likely to abuse children or to show paraphilic interests.

Logistic regressions revealed that selected RSVP items allowed predicting group membership (MIQSO vs. MSO) with 37% of the variance explained. The best predictors were escalation of sexual violence and psychological coercion in sexual violence for MSO and problems with planning for MIQSO. Escalation of sexual violence refers to an increase in number, intensity or gravity of sexual violence (17, 94). In other words, MSO are more likely to show some characteristics associated with mainstream (or paraphilic) sexual recidivism than MIQSO, who are less likely to have a history of sexual violence or to aggravate their behavior. Psychological coercion refers to using a position of power and authority to gain compliance from a victim who is dependent or vulnerable, which may include children (17). Therefore, the nature of index crime (indecent contact behavior), the trend toward more sexual deviancy, and this result suggest that MIQSO could be less likely to victimize children than MSO. It would be interesting to investigate this trend in future research. Importantly, however, if confirmed this result should not be interpreted as evidence against an association between IQ and victim age among men who have sexually offended. The mean IQ of the MSO in this study was still in the low average range (86 ± 13.8), comparable to that reported in studies finding a link between lower IQ and apprehended men with pedophilia [e.g., (111)]. Therefore, lower IQ should not be confounded with low IQ (<70). The possibility remains that the association between IQ and apprehended pedophiles is not linear, perhaps vanishing below a certain point (e.g., <70). Given that official legal records in Belgium do not systematically specify the age of the victim (e.g., rape, violent indecent contact behavior), it was not possible to assess the link between IQ and victim age in this study. Further investigation with MIQSO should verify the linearity of the association between IQ and victim age. It would also be interesting to include the other end of the spectrum, that is, men with significantly higher IQ (13).

Overall, these results suggest that few but important differences exist for risk factors associated with forensic clients who have sexually offended as a function of their IQ level. Whereas for those with an average IQ classic risk factors associated with general delinquency (e.g., substance abuse, nonviolent criminality) and mainstream (or paraphilic) sexual recidivism (e.g., chronicity, coercion, escalation) are important, for those with a low IQ dynamic factor associated with general psychiatry are more significant (e.g., lack of insight, awareness, and sexual knowledge; contextual impulsivity).

Still, these results should be interpreted with caution given the study limits. First, inter-group comparisons were exploratory, based on relatively small sample sizes and a series of uncorrected bivariate statistics, which might have inflated the number of significant differences. Consideration for effect sizes is warranted to interpret these results. Second, lack of access to materials or training at the beginning of data collection prevented assessing all participants with phallometry and psychopathy (PCL-R) measures. Adding these two important measures of the SORAG might have generated different or refined results. Third, the SORAG and RSVP were used for research purposes, based on their single items and factors. Although total scores are provided, they should not be interpreted as evaluations of risk to recidivate. Likewise, only one assessment of the RSVP was conducted in this study, although this instrument was developed for repeated measures of the dynamic factors (fluctuating variables). Fourth, study groups were divided on the sole basis of their IQ, without assessment of adaptive behaviors because no French validated instruments were available at the time of data collection and this type of assessment is complex within secure environments (112). Fifth, these results were obtained with relatively old participants (mostly in their forties), so that confirmation is warranted with younger participants (and larger sample size). Sixth, some risk factors relevant to MIQSO not included in the measures were not considered in this study, such as low self-esteem, cognitive distortions, chaotic family background, and lack of attachment bounds (19, 20). Finally, and most importantly, crucial outcome variables such as recidivism and treatment completion rates were not available in this cross-sectional study, limiting the predictive value of the results. Future investigations should consider these additional factors.

## Conclusions

Although individuals with a low IQ or ID are commonly thought to be at higher risk to commit an offense, evidence-based data are still lacking and difficult to obtain. In order to avoid further stigmatization of persons with a low IQ, it is of upmost importance to better understand the origins and context associated with violent and sexual offenses committed by a minority. Risk assessments in forensic settings for persons with a low IQ would allow clinicians to better determine who can be discharged and the particular needs of those at higher risks to re-offend. Promising risk assessment tools devoted to persons with ID slowly begin to emerge (e.g., ARMIDILO-S) and should be translated and validated in other languages (e.g., in French). In the meantime, this study suggest that sexual offenses committed by forensic patients with a low IQ have, on average, different clinical and criminological origins than those committed by forensic patients with an average IQ.

## Data Availability Statement

The raw data supporting the conclusions of this article will be made available by the authors, without undue reservation.

## Ethics Statement

The studies involving human participants were reviewed and approved by Hôpital Les Marroniers. The patients/participants provided their written informed consent to participate in this study.

## Author Contributions

TP planned and supervised the study. AV and ÉT collected the data. AV, ÉT, and CJ analyzed the data. CJ and AV wrote the manuscript. TP and ÉT reviewed the manuscript. All authors contributed to the article and approved the submitted version.

## Funding

CJ received a research grant from the Office des personnes handicapées du Québec (OPHQ; Ref. 2231), which had no direct involvement in the research or preparation of the manuscript.

## Conflict of Interest

The authors declare that the research was conducted in the absence of any commercial or financial relationships that could be construed as a potential conflict of interest.

## Publisher's Note

All claims expressed in this article are solely those of the authors and do not necessarily represent those of their affiliated organizations, or those of the publisher, the editors and the reviewers. Any product that may be evaluated in this article, or claim that may be made by its manufacturer, is not guaranteed or endorsed by the publisher.
